# Bilaterally Diffuse Lacrimal Gland Involvement: Initial Presentation of Systemic Sarcoidosis

**DOI:** 10.4274/tjo.89310

**Published:** 2017-06-01

**Authors:** Pınar Bingöl Kızıltunç, Fatma Çiftçi, Banu Hoşal, Gülşah Kaygusuz

**Affiliations:** 1 Kağızman State Hospital, Ophthalmology Clinic, Kars, Turkey; 2 Ankara University Faculty of Medicine, Department of Chest Diseases, Ankara, Turkey; 3 Ankara University Faculty of Medicine, Department of Ophthalmology, Ankara, Turkey; 4 Ankara University Faculty of Medicine, Department of Pathology, Ankara, Turkey

**Keywords:** Lacrimal gland, Orbit, Sarcoidosis

## Abstract

Orbital involvement in systemic sarcoidosis is a rare condition. We report a case of orbital sarcoidosis with bilaterally huge lacrimal gland involvement as the initial manifestation of systemic sarcoidosis. A 20-year-old woman admitted the ophthalmology department with progressive bilateral upper eyelid swelling for 6 months. The only pathologic finding was the presence of bilateral, symmetrical, solid, lobular masses at the lateral upper eyelids at the location of lacrimal glands. On systemic examination, bilateral parotid and submandibular glands appeared swollen. Magnetic resonance imaging of the orbit revealed bilateral symmetrical diffuse enlargement of the lacrimal glands with maximum and minimum thickness of 11 mm and 7 mm, respectively. The biopsy findings were compatible with sarcoidosis. Although lacrimal gland involvement has been reported in different studies, we for the first time report an unusual case with bilateral diffuse huge lacrimal gland involvement. Normal lacrimal gland thickness is approximately 4-5 mm in magnetic resonance imaging, while our case had bilateral diffuse enlargement of lacrimal glands, which showed maximum and minimum thickness of 11 mm and 7 mm, respectively. Although orbital involvement is uncommon in sarcoidosis, it should be remembered in the differential diagnosis of orbital masses.

## INTRODUCTION

Sarcoidosis is an idiopathic, multisystem disorder that can affect any organ system and is mainly characterised by pulmonary, dermatologic, and ocular involvement. Its pathological hallmark is non-caseating granulomatous inflammation. Ocular involvement has been reported by different studies at a rate of 25-60%.^1,2^ Although anterior uveitis is the most common manifestation of ocular sarcoidosis, any orbital structure can be involved. Lacrimal gland involvement is the most common form of orbital sarcoidosis.^1,3^ We present a case of orbital sarcoidosis with bilateral enlargement of the lacrimal glands with eyelid and anterior orbital involvement as the initial manifestation of systemic sarcoidosis.

## CASE REPORT

**Figure 1:**
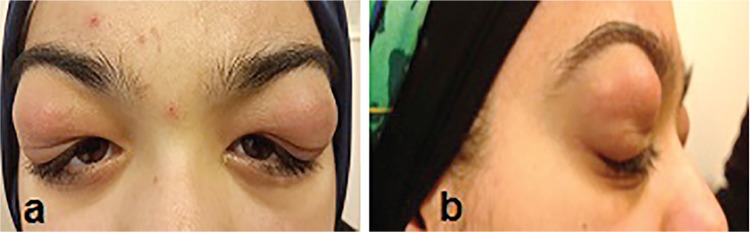
a, b) Anterior and lateral views showing solid, indurated, lobulated masses at the lateral parts of both upper eyelids corresponding to the location of the lacrimal glands

A 20-year-old woman was admitted to the ophthalmology department with progressive bilateral upper eyelid swelling for 6 months. She had no other symptoms related to her eyes. A physical examination revealed dry mouth and nasal congestion. She had a history of triamcinolone (Nasacort) nasal spray usage for nasal congestion for nine months. Her family history was unremarkable. Her best corrected visual acuity was 10/10 in both eyes. The only pathologic finding identified through slit-lamp biomicroscopy was the presence of bilateral, symmetrical, solid, lobular masses in the lateral upper eyelids at the location of the lacrimal glands (Figure 1a). There was no proptosis. The patient’s dilated fundus examination was unremarkable. Intraocular pressure was measured as 16 mmHg in both eyes. Pupillary response to light and eye movements was normal. The result of a Schirmer test without anaesthesia was 1 mm/5 minutes in both eyes.

**Figure 2:**
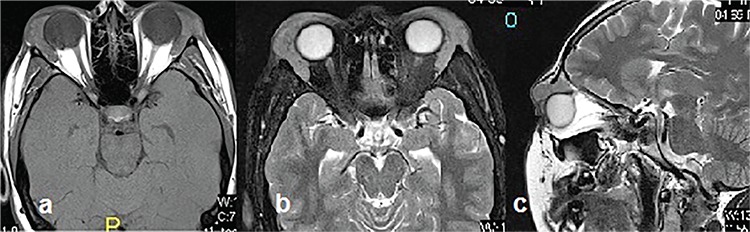
Magnetic resonance imaging showed bilateral symmetrical diffuse enlargement of the lacrimal glands and involvement of the superior eyelids and anterior orbit: isointense signal intensity relative to muscle on T1-weighted axial image (a); hypointense signal intensity on T2-weighted axial image (b); hypointense signal intensity on T2-weighted sagittal image (c)

**Figure 3:**
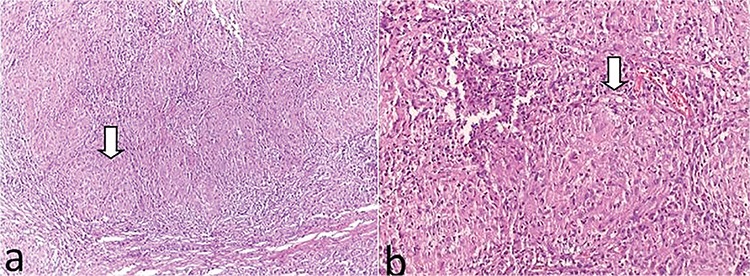
a, b) Left lacrimal gland biopsy showed discrete granulomas (arrow) (hematoxylin-eosin, x100, x200)

Skin examination revealed subcutaneous nodules in the scalp. Upon systemic examination, the bilateral parotid and submandibular glands appeared swollen (Figure 1b). Magnetic resonance imaging (MRI) of the orbit revealed involvement of the superior eyelids and the anterior orbit and bilateral symmetrical diffuse enlargement of the lacrimal glands with an isointense signal intensity relative to muscle on T1-weighted images and a hypointense signal intensity on T2-weighted images (Figure 2). On MRI, the maximum and minimum thicknesses of the lacrimal glands were 11 mm and 7 mm, respectively. Parotid and submandibular glands were evaluated with ultrasound and MRI. Neck ultrasonography showed heterogeneous and hypoechoic areas in the parotid and submandibular glands bilaterally. MRI of the neck showed bilateral cervical lymph nodes of pathological size and bilateral enlargement of the parotid and submandibular glands with a heterogeneous appearance. For definitive diagnosis, a lacrimal gland biopsy was taken from the orbital lobe using an upper lid crease incision. Microscopic examination showed discrete non-necrotising granulomas (Figure 3). Acid fast bacilli were not identified by Ehrlich-Ziehl-Neelsen staining. Lymphoma was not considered in the differential diagnosis because of the absence of numerous atypical lymphocytes. The biopsy findings were consistent with sarcoidosis.

The patient was referred to the chest disease department for pulmonary involvement. Laboratory examination showed an elevated angiotensin converting enzyme level of 63 U/L. Blood and urine calcium levels were within normal limits. The tuberculin skin test result was anergic. A chest x-ray demonstrated bilateral hilar enlargement. A thoracic computer tomography revealed bilateral hilar, subcarinal, and aortopulmonary lymphadenopathies as well as perilymphatic and peribronchovascular nodules in both lungs. Pulmonary function test results (maximal expiratory flows with spirometry and diffusion capacity test) were normal. No treatment was recommended for the pulmonary involvement.

**Figure 4:**
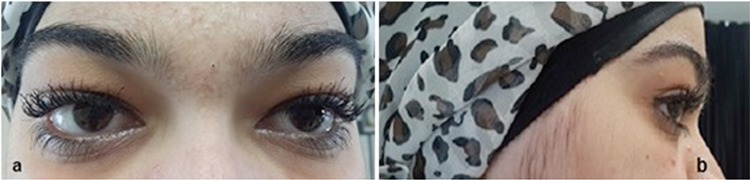
a, b) Marked regression in the ocular lesions is evident in anterior (a) and lateral (b) views after 1 year of treatment

Due to the enlarged lacrimal glands and the eyelid and anterior orbital involvement affecting the patient’s visual capacity, oral methylprednisolone 0.5 mg/kg/day was prescribed. Symptomatic improvement soon became evident, and at the 21^st^ day of treatment the steroid dose was reduced to 4 mg/2 weeks. The patient was treated with tapered dose steroids for nine months, and no relapse was observed at the first year follow-up (Figure 4). After the treatment, the Schirmer test result without anaesthesia was 4 mm/5 minutes in both eyes.

## DISCUSSION

Ocular adnexal sarcoidosis usually presents as a local mass. We present an unusual bilateral enlargement of the lacrimal glands and involvement of the anterior orbit and eyelids due to orbital sarcoidosis as the initial manifestation of systemic sarcoidosis.

Lacrimal glands are the most commonly involved structures of the orbit in orbital sarcoidosis.^3,4,5^ The prevalence of lacrimal gland involvement varies across studies due to varying diagnostic criteria. Two large studies reported lacrimal gland involvement at rates of 7% and 15.8%.^2,6^ These studies based the diagnosis of orbital sarcoidosis on lacrimal gland enlargement and the presence of dry eye symptoms. However, sarcoidosis is a pathologic diagnosis, so a biopsy is recommended for a definitive diagnosis.

Because of the inflammatory nature of sarcoidosis, orbital symptoms usually mimic other inflammatory diseases that involve orbital structures. Sjögren’s syndrome, tuberculosis, lymphoma and immunoglobulin G4 (IgG4)-related Mikulicz’s disease are the main pathologies that should be considered in the differential diagnosis of sarcoidosis. Although these diseases can be seen at any age, Sjögren’s syndrome and tuberculosis are the primary diseases for the differential diagnosis of sarcoidosis in younger patients. These diseases can cause bilateral involvement and are usually characterised by painless enlargement of lacrimal glands for more than one month. Although clinical findings and imaging tests can help guide clinicians, a biopsy is required for all patients with orbital masses of unknown origin.

The characteristic histological feature of sarcoidosis is non-caseating granulomas consisting of epithelioid histiocytes and lymphocytes. Multinucleated giant cells are frequently seen. Although tuberculosis is also characterised by chronic granulomatous inflammation, in tuberculosis the granulomas tend to be coalescent with necrosis. The presence of atypical lymphocytes in lymphoma, IgG4-positive plasma cells in IgG4-related Mikulicz’s disease, periductal and perivascular inflammation of lymphocytes and intralobular fibrosis in Sjögren’s syndrome are the main factors that aid in the differential diagnosis of sarcoidosis.^7^

This case is important because the first symptom of systemic sarcoidosis in this case was diffuse enlargement of the lacrimal glands with eyelid and anterior orbital involvement. MRI reveals normal lacrimal gland thickness to be approximately 4-5 mm,^8^ whereas our case had bilateral diffuse enlargement of the lacrimal glands, which possessed maximum and minimum thicknesses of 11 mm and 7 mm, respectively.

Although orbital involvement is uncommon in sarcoidosis, it should be considered in the differential diagnosis of orbital masses. This case is striking compared to the previous case reports in the literature with respect to bilateral and substantially larger lacrimal gland involvement. The diagnosis of sarcoidosis should be made by clinical, laboratory, and radiological findings and confirmed by histopathological examination. It is necessary to screen all systems, and treatment decisions should be based on the presence of the organ and system involvement.
